# A nomogram based on nutritional and inflammatory parameters to predict DMFS and identify beneficiaries of adjuvant chemotherapy in IVA-stage nasopharyngeal carcinoma

**DOI:** 10.1186/s12885-024-12330-6

**Published:** 2024-05-11

**Authors:** Yuhui pan, Zihan Chen, Wenquan Hong, Zongwei Huang, Ying Li, Sunqin Cai, Jinghua Lai, Jun Lu, Sufang Qiu

**Affiliations:** 1 Department of Radiation Oncology, Clinical Oncology School of Fujian Medical University, Fujian Cancer Hospital (Fujian Branch of Fudan University Shanghai Cancer Center), Fuzhou, Fujian 350014 China; 2 Clinical Oncology School of Fujian Medical University, Fujian Cancer Hospital (Fujian Branch of Fudan University Shanghai Cancer Center), Fuzhou, Fujian China

**Keywords:** Nasopharyngeal carcinoma, Nomogram, S-1 adjuvant chemotherapy, Risk stratification, Prognosis

## Abstract

**Objective:**

This study aims to develop a nomogram integrating inflammation (NLR), Prognostic Nutritional Index (PNI), and EBV DNA (tumor burden) to achieve personalized treatment and prediction for stage IVA NPC. Furthermore, it endeavors to pinpoint specific subgroups that may derive significant benefits from S-1 adjuvant chemotherapy.

**Methods:**

A total of 834 patients diagnosed with stage IVA NPC were enrolled in this study and randomly allocated into training and validation cohorts. Multivariate Cox analyses were conducted to identify independent prognostic factors for constructing the nomogram. The predictive and clinical utility of the nomogram was assessed through measures including the AUC, calibration curve, DCA, and C-indexes. IPTW was employed to balance baseline characteristics across the population. Kaplan-Meier analysis and log-rank tests were utilized to evaluate the prognostic value.

**Results:**

In our study, we examined the clinical features of 557 individuals from the training cohort and 277 from the validation cohort. The median follow-up period was 50.1 and 49.7 months, respectively. For the overall cohort, the median follow-up duration was 53.8 months. The training and validation sets showed 3-year OS rates of 87.7% and 82.5%, respectively. Meanwhile, the 3-year DMFS rates were 95.9% and 84.3%, respectively. We created a nomogram that combined PNI, NRI, and EBV DNA, resulting in high prediction accuracy. Risk stratification demonstrated substantial variations in DMFS and OS between the high and low risk groups. Patients in the high-risk group benefited significantly from the IC + CCRT + S-1 treatment. In contrast, IC + CCRT demonstrated non-inferior 3-year DMFS and OS compared to IC + CCRT + S-1 in the low-risk population, indicating the possibility of reducing treatment intensity.

**Conclusions:**

In conclusion, our nomogram integrating NLR, PNI, and EBV DNA offers precise prognostication for stage IVA NPC. S-1 adjuvant chemotherapy provides notable benefits for high-risk patients, while treatment intensity reduction may be feasible for low-risk individuals.

**Supplementary Information:**

The online version contains supplementary material available at 10.1186/s12885-024-12330-6.

## Introduction

Nasopharyngeal Carcinoma (NPC), a malignant tumor of the head and neck originating from nasopharyngeal epithelium, exhibits distinct epidemiological features, with the highest incidence observed in Southern China and Southeast Asia [1]. In 2020, the global incidence of NPC accounted for approximately 133,000 new cases and 80,000 fatalities, with China representing nearly half of these cases [2]. Presently, prognosis remains dismal for locally advanced stages, particularly for stage IVA NPC, with a 5-year OS rate lingering around 65% [3]. 19–29% of NPC patients develop distant metastasis post-treatment, a figure that escalates in stage IVA [4]. Distant metastasis has emerged as a predominant cause of treatment failure in NPC [5, 6].

Despite strides in immunotherapy and platinum-based chemotherapy for metastatic NPC, the prognosis for patients with metastases remains bleak, with a median OS of 20–29 months [7, 8]. Consequently, stratifying the risk of distant metastasis is vital, especially in stage IVA NPC, to facilitate targeted interventions.

The TNM staging system is pivotal in prognostication and treatment guidance for cancer patients, yet the role of pre-treatment haematological indicators is increasingly recognized. Plasma EBV DNA has emerged as a reliable biomarker for NPC, instrumental in diagnosis, treatment planning, risk stratification, prognostic evaluation, and condition monitoring [9, 10]. Pre-treatment plasma EBV DNA levels have been identified as predictors of distant metastasis in NPC [11].

The Prognostic Nutritional Index (PNI) serves as a straightforward and practical indicator for predicting the nutritional status of cancer patients [12]. Meanwhile, NLR represents inflammatory markers [13–15]. Recently, emerging indicators such as PNI and NLR have garnered significant attention in prognosticating the outcomes of nasopharyngeal carcinoma (NPC) patients. Moreover, their effectiveness has been validated by previous research endeavors [16].

However, comprehensive research on tumor burden, nutrition, and inflammation prognostic markers for stage IVA NPC, as well as the establishment of an effective distant metastasis prediction model, are still lacking. Additionally, many patients undergoing concurrent chemoradiotherapy (CCRT) may experience severe acute toxic reactions, leading to reduced compliance with subsequent adjuvant chemotherapy (AC). Of note, S-1, an oral fluoropyrimidine, has shown potential in improving overall survival and metastasis-free survival in previous studies [17, 18]. Therefore, this study aims to establish effective prognostic indicators from the perspective of reducing toxicity and achieving personalized treatment, identifying high-risk patients most likely to benefit from adjuvant S-1 therapy.

## Methods

### Study population

This retrospective study analyzed patients diagnosed with stage IVA NPC at Fujian Cancer Hospital from July 2016 to December 2019. Inclusion criteria were: (1) histological diagnosis of squamous cell carcinoma; (2) stage IVA as per the 8th edition UICC/AJCC classification; (3) treatment with IMRT; (4) complete baseline clinical and laboratory data; and (5) comprehensive follow-up records. Exclusion criteria included: (1) distant metastasis at diagnosis; (2) prior anti-tumor treatment; and (3) comorbidities significantly affecting complete blood count or biochemistry, such as aplastic anemia, myelofibrosis, acute or chronic hepatitis, cirrhosis, etc. A total of 834 patients met these criteria. The study adhered to the Helsinki Declaration, was approved by our institutional ethics committee, and all patients provided written informed consent before treatment.

### Treatment protocol

All patients received standardized treatment as per NPC guidelines, including induction chemotherapy (IC), IMRT, concurrent chemotherapy (CC), adjuvant chemotherapy (AC), and targeted therapy.IC: Comprised of platinum-based drugs combined with taxanes, doxorubicin, 5-fluorouracil, or gemcitabine, administered intravenously every three weeks for 1–7 cycles.CC: Single-drug platinum-based chemotherapy intravenously every three weeks for 1–3 cycles.AC: Oral maintenance therapy with Teysuno (S-1) or capecitabine, administered every four weeks for at least two cycles.Targeted Therapy: Nimotuzumab (NTZ) or Endostar (E), or their combination, primarily during IC and/or radiotherapy. NTZ was given intravenously at 200 mg/week for 3–21 cycles, and E was administered at 7.5 mg/m2 on days 1–14, every three weeks for 2–8 cycles. Specific treatment details are available in Additional file 1.

### Follow-up and study endpoints

Post-treatment, patients were followed every three months for the first two years, every six months for years 2–5, and annually thereafter until death. Routine physical examinations, nasopharyngeal endoscopy, nasopharyngeal and neck MRI, abdominal ultrasound, chest CT, plasma EBV DNA level measurements, and other hematologic markers were monitored. PET-CT was considered when necessary. The primary endpoint was distant metastasis-free survival (DMFS); the secondary endpoint was OS. DMFS was defined as the time from diagnosis to the first occurrence of distant metastasis, death from any cause, or the last follow-up, whichever occurred first. OS was measured from the date of diagnosis to death from any cause or the last follow-up.

### Statistical analysis

**Fig. 1:**
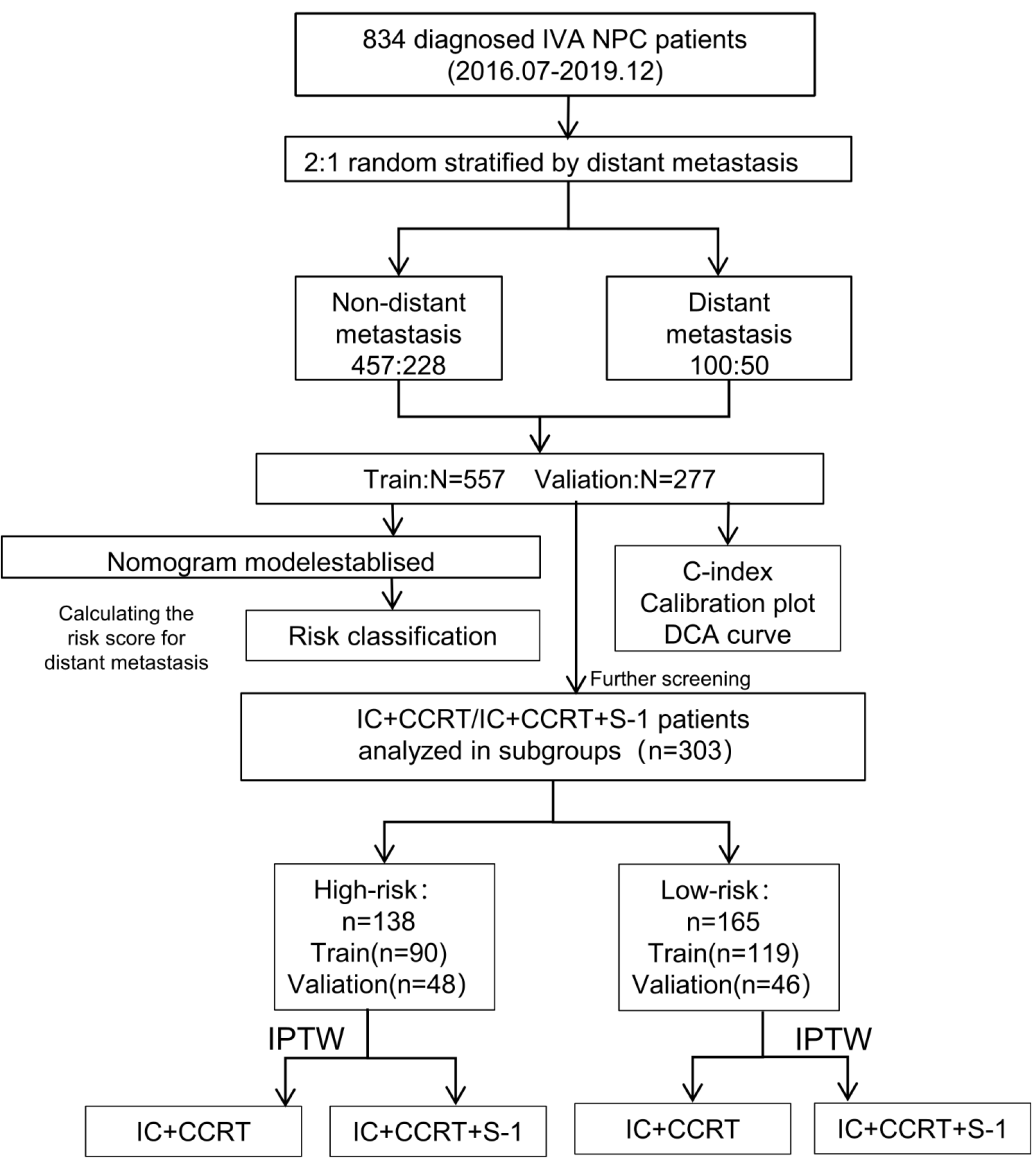
The flowchart of the enrolled patients

Statistical analysis was performed using R language (version 4.2.2). Using SPSS 26 software, patients were stratified based on the presence or absence of distant metastasis. Subsequently, a 2:1 randomized stratified sampling approach was employed to divide the patients into training and validation sets. This process is graphically represented in Fig. 1. Maximally Selected Rank Statistics’ optimum cutoff value was used to dichotomize the candidate continuous variables. Variables with a P-value < 0.05 in the baseline table entered the multivariate logistic regression [19]to assess independent prognostic factors for distant metastasis. We developed a predictive model based on multivariate analysis and clinically relevant factors, assigning scores to each factor in the model based on its association with the risk of distant metastasis in nasopharyngeal carcinoma patients. Patients were then stratified into high-risk and low-risk groups accordingly. Utilizing the tree model and the “partykit” package, we can accurately stratify nomogram scores into precise subgroups. Decision curve analysis (DCA) was performed to determine the clinical utility of the nomogram, quantifying net benefits at different probability thresholds in the training cohort. Survival outcomes were analyzed using the Kaplan-Meier method, with survival curves compared using the log-rank test. In addition, Inverse Probability of Treatment Weighting (IPTW) was utilized to balance baseline characteristics, checking for standardized mean differences (SMD) < 0.1. All statistical tests were two-sided, and a P-value < 0.05 was considered statistically significant.

## Results

### Clinical characteristics and survival outcomes

We analyzed the clinical profiles of 557 patients in the training cohort and 277 in the validation cohort (Table 1). Overall, they exhibited comparable characteristics. The median age for both groups was 48 years. The median follow-up time for the entire cohort was 53.8 months (95% CI 52.9–55). Among the study population, 150 stage IVA patients developed distant metastases: 90 had single-organ involvement, and 60 had multiple metastases, primarily in the bone, lung, and liver. Notably, 18% of patients in each cohort experienced distant metastases (100 in the training cohort and 50 in the validation cohort). In the validation set, the 3-year OS rate was 82.5% (95% CI = 0.767–0.877), while the 3-year DMFS rate was 95.9% (95% CI = 0.924–0.995). For the training set, the 3-year OS rate was 87.7% (95% CI = 0.850–0.905), and the 3-year DMFS rate was 84.3% (95% CI = 0.813–0.874). The overall 3-year OS rate was 88.0% (95% CI = 0.858–0.902), and the overall 3-year DMFS rate was 84.1% (95% CI = 0.816–0.867).

**Table 1:** Clinical characteristics of patients in training cohort and validation cohort

	No distant metastases (training cohort) n = 457	Distant metastases (training cohort) n = 100	*P* value	No distant metastases (validation cohort) n = 227	Distant metastases (validation cohort) n = 50	*P* value
Gender			0.383			0.444
Female	110 (24.1%)	20 (20%)		52 (22.9%)	14 (28%)	
Male	347 (75.9%)	80 (80%)		175 (77.1%)	36 (72%)	
Age (years)			0.367			0.244
<60	361 (79%)	83 (83%)		184 (81.1%)	44 (88%)	
≥ 60	96 (21%)	17 (17%)		43 (18.9%)	6 (12%)	
Pathological type			0.576			0.151
WHO I	5(1.1%)	0(0%)		3 (1.3%)	0 (0%)	
WHO II	27(5.9%)	6(6%)		20 (8.8%)	9 (18%)	
WHO III	425(93%)	94(94%)		204 (89.9%)	41 (82%)	
T stage			0.272			0.721
0	2 (0.4%)	0 (0.0%)		0 (0.0%)	0 (0.0%)	
1	46 (10.1%)	15 (15.0%)		21 (9.3%)	6(12%)	
2	55 (12.0%)	13 (13.0%)		25 (11%)	6(12%)	
3	64 (14.0%)	19 (19.0%)		35 (15.4%)	10 (20%)	
4	290 (63.7%)	53 (53.0%)		146 (64.3%)	28(56%)	
N stage			<0.001			0.005
0	24 (5.3%)	0 (0%)		9(4%)	1(2%)	
1	135 (29.5%)	12 (12%)		75(33%)	5 (10%)	
2	104 (22.8%)	31 (31%)		57(25.1%)	14(28%)	
3	194(42.5%)	57 (57%)		86(37.9%)	30(60%)	
pre LDH (U/L)			0.024			0.008
< 204	347(76.0%)	65 (65%)		184 (81.06)	32 (64.00)	
≥ 204	110 (24.7%)	35 (35%)		43 (18.94)	18 (36.00)	
pre EBV DNA (copies/mL)			<0.001			< 0.001
< 10,100	282 (61.7%)	34 (34%)		133 (58.6%)	15 (30%)	
≥ 10,100	175((38.3%)	66 (66%)		94 (41.4%)	35 (70%)	
White blood cells, **10**^**9**^**/L**			0.570			0.190
< 7.4	261 (57.1%)	54 (54%)		132 (58.1%)	24 (48.0%)	
≥ 7.4	196 (42.9%)	46 (46%)		95 (41.9%)	26 (52.0%)	
Platelet count, **10**^**9**^**/L**			0.186			0.749
<306	336 (73.5%)	67 (67%)		164 (72.2%)	35 (70.0%)	
≥ 306	121 (26.5%)	33 (33%)		63 (27.8%)	15 (30.0%)	
Neutrophil count, **10**^**9**^**/L**			0.586			0.198
<4.1	259 (56.7%)	45 (45.0%)		95 (41.8%)	16 (32.0%)	
≥ 4.1	198 (43.3%)	55 (55.0%)		132 (58.2%)	34 (68.0%)	
PNI			0.065			0.225
<57.9	377 (82.5%)	90 (90.0%)		200 (88.1%)	47 (94.0%)	
≥ 57.9	80 (17.5%)	10 (10.0%)		27 (11.9%)	3 (6.0%)	
NLR			0.034			0.018
<2.32	259 (56.7%)	45 (45.0%)		128 (56.4%)	19 (38.0%)	
≥ 2.32	198 (43.3%)	55 (55.0%)		99 (43.6%)	31 (62.0%)	
PLR			0.163			0.013
< 196.5	395 (86.4%)	81 (81.0%)		196 (86.3%)	36 (72.0%)	
≥ 196.5	62 (13.6%)	19 (19.0%)		31 (13.7%)	14 (28.0%)	
AGR			0.528			0.258
< 1.2	119 (26.0%)	23 (23.0%)		51 (22.5%)	15 (30.0%)	
≥ 1.2	338 (74.0%)	77 (77.0%)		176 (77.5%)	35 (70.0%)	
ALP, **U/L**			0.388			0.110
< 101	304 (66.5%)	62 (62.0%)		154 (67.8%)	28 (56.0%)	
≥ 101	153 (33.5%)	38 (38.0%)		73 (32.2%)	22 (44.0%)	
FIB, **g/L**			0.257			0.042
< 3.05	202 (44.2%)	38 (38.0%)		99 (43.6%)	14 (28.0%)	
≥ 3.05	255 (55.8%)	62 (62.0%)		128 (56.4%)	36 (72.0%)	
treatment paradigm			0.423			0.898
IC + RT	26 (5.7%)	4 (4%)		13 (5.7%)	2 (4%)	
IC + RT + NTZ/E	51 (11.1%)	10 (10.1%)		32 (14.1%)	5 (10%)	
IC + RT + AC	17 (3.7%)	6 (6.1%)		9 (4%)	4 (8%)	
IC + RT + AC + NTZ/E	55 (12%)	9 (9.1%)		31 (13.7%)	6 (12%)	
IC + CCRT	128 (28%)	26 (35%)		60 (26.4%)	13 (26%)	
IC + CCRT + NTZ/E	31 (6.8%)	11 (11.1%)		25 (11%)	5 (10%)	
IC + CCRT + AC	76 (16.6%)	13 (13%)		27 (11.9%)	8 (16%)	
IC + CCRT + AC + NTZ/E	73 (16%)	12 (12%)		30 (13.2%)	7 (14%)	

IC + RT: Induction chemotherapy followed by radiotherapy.IC + RT + NTZ/E: Induction chemotherapy and radiotherapy, augmented with Nitolizumab/Endostar.IC + RT + AC: Induction chemot-herapy, radiotherapy and adjuvant chemotherapy.IC + RT + AC + NTZ/Endostar: Induction chemotherapy, radiotherapy, adjuvant chemotherapy, and nitolizumab/Endostar.IC + CCRT: Inductio-n chemotherapy combined with concurrent radiotherapy.IC + CCRT + NTZ/Endostar: Induction chemotherapy, concurrent radiotherapy and nitolizumab/Endostar.IC + CCRT + AC: Induction c-hemotherapy, concurrent radiotherapy and adjuvant chemotherapy.IC + CCRT + AC + NTZ/Endost-ar: Induction chemotherapy, concurrent radiotherapy, adjuvant chemotherapy, and nitolizum-ab/Endostar.AGR: Albumin to Globulin ratio.ALP: alkaline phosphatase.FIB: Fibrinogen

### Development and validation of the nomogram

**Fig. 2:**
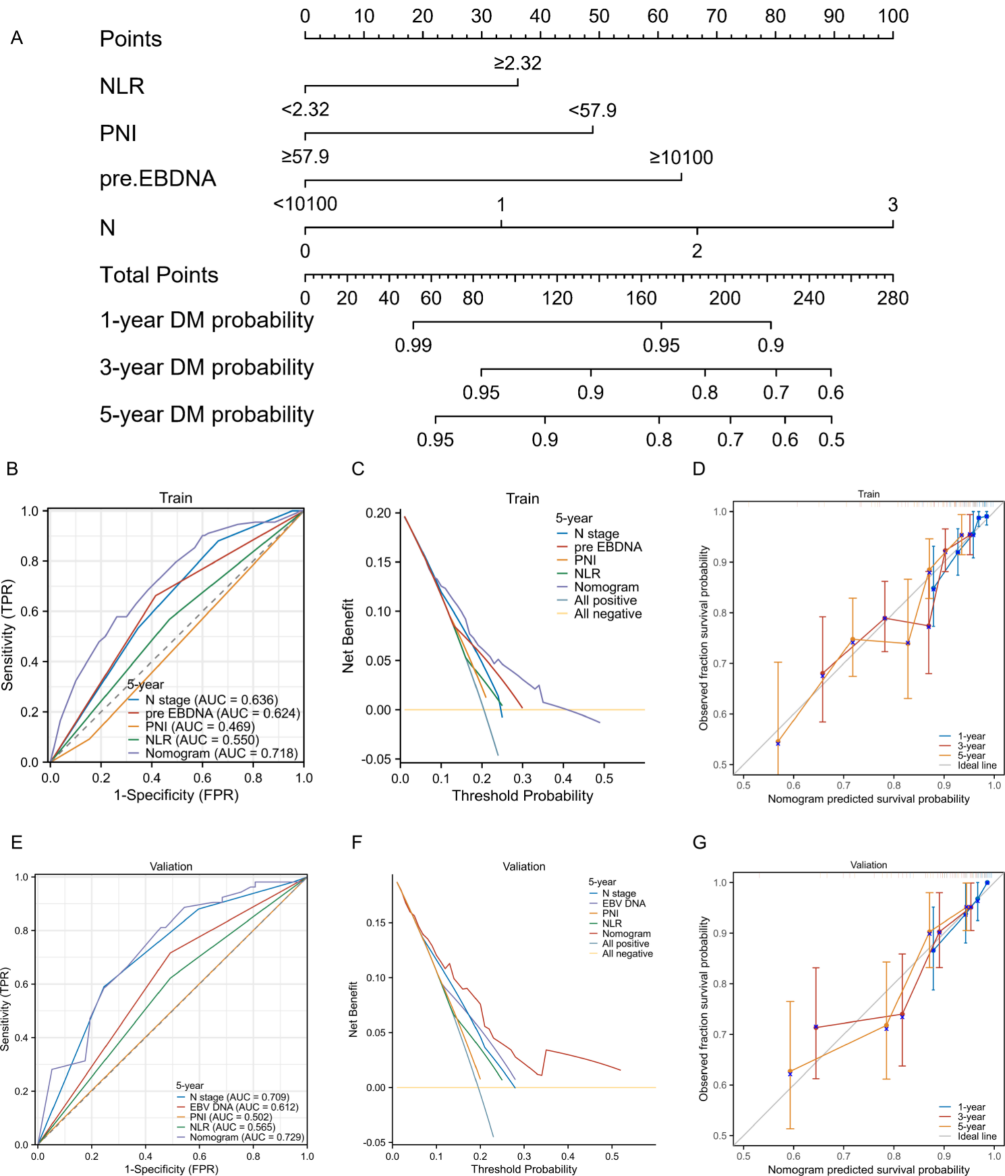
Nomogram Construction for DMFS in IVA NPC (**A**). AUC of the nomogram at 5-year in the training set (**B**) and validation set (**E**). Decision curves analysis of the nomogram at 5 years in the training set (**C**) and validation set (**F**).Calibration plot of the nomogram at 1-, 3-, and 5-year in the training set (**D**) and validation set (**G**). AUC, area under curve; pre EBV DNA: pre-treatment EBV DNA; NLR: Neutrophil to Lymphocyte Ratio; DMdistant metastasis; PNI: prognostic nutritional index

In the training set, both univariate and multivariate Cox regression models are presented in Table 2. The results of the multivariate Cox regression model indicate correlations between PNI, NRI, N stage, EBV DNA. Based on these four independent prognostic factors, a nomogram model was developed, illustrated in Fig. 2.The area under the receiver operating characteristic (ROC) curve (AUC) for the model demonstrated good predictive accuracy, with an AUC of 0.718 (95% CI = 0.650–0.785) in the training cohort (Fig. 2A) and 0.729 (95% CI = 0.635–0.823) in the validation cohort (Fig. 2B). Calibration curves for both the training and validation cohorts (Fig. 2D and G) closely aligned with the diagonal line, indicating a high concordance between the model’s predictions and the actual outcomes for patients. The AUC values for all independent factors are presented in Fig. 2B and E, showing that in both the training and validation cohorts, the nomogram achieved the highest AUC. We constructed decision curve analysis (DCA) curves for the nomogram and each independent factor, as depicted in Fig. 2C and F. The net benefit of the nomogram surpassed that of individual independent factors significantly.

**Table 2:** Univariate and multivariate cox proportional hazards models of distant metastasis

Variables	Univariate						Multivariate				
	β	S.E	Z	*P*	HR (95%CI)		β	S.E	Z	*P*	HR (95%CI)
T											
0–1					1.00 (Reference)						1.00 (Reference)
2	-0.31	0.38	-0.82	0.413	0.73 (0.35 ~ 1.54)		-0.30	0.38	-0.79	0.431	0.74 (0.35 ~ 1.56)
3	-0.10	0.35	-0.28	0.778	0.91 (0.46 ~ 1.79)		-0.31	0.35	-0.87	0.386	0.74 (0.37 ~ 1.47)
4	-0.53	0.29	-1.81	0.071	0.59 (0.33 ~ 1.05)		-0.23	0.39	-0.57	0.567	0.80 (0.37 ~ 1.73)
N											
0–1					1.00 (Reference)						1.00 (Reference)
2	1.19	0.34	3.48	< 0.001	3.27 (1.68 ~ 6.37)		0.99	0.34	2.88	0.004	2.69 (1.37 ~ 5.28)
3	1.23	0.32	3.87	< 0.001	3.41 (1.83 ~ 6.37)		1.08	0.42	2.58	0.010	2.95 (1.30 ~ 6.71)
Gender											
Female					1.00 (Reference)						
Male	0.20	0.25	0.82	0.412	1.23 (0.75 ~ 2.00)						
Age (years)											
<60					1.00 (Reference)						
≥ 60	-0.13	0.27	-0.50	0.618	0.88 (0.52 ~ 1.48)						
pre LDH (U/L)											
< 204					1.00 (Reference)						1.00 (Reference)
≥ 204	0.48	0.21	2.27	0.023	1.61 (1.07 ~ 2.43)		0.28	0.22	1.27	0.205	1.32 (0.86 ~ 2.03)
pre EBV DNA (copies/mL)											
< 10,100					1.00 (Reference)						1.00 (Reference)
≥ 10,100	0.99	0.21	4.68	< 0.001	2.69 (1.78 ~ 4.06)		0.85	0.22	3.92	< 0.001	2.34 (1.53 ~ 3.58)
White blood cells, 109/L											
< 7.4					1.00 (Reference)						
≥ 7.4	0.14	0.20	0.70	0.482	1.15 (0.78 ~ 1.71)						
Platelet count, 109/L											
<306					1.00 (Reference)						
≥ 306	0.31	0.21	1.44	0.150	1.36 (0.90 ~ 2.06)						
PNI											
<57.9					1.00 (Reference)						1.00 (Reference)
≥ 57.9	-0.61	0.33	-1.83	0.068	0.54 (0.28 ~ 1.05)		-0.69	0.35	-2.00	0.045	0.50 (0.25 ~ 0.99)
Neutrophil count, 109/L											
<4.1					1.00 (Reference)						
≥ 4.1	0.13	0.20	0.64	0.521	1.14 (0.76 ~ 1.70)						
NLR											
<2.32					1.00 (Reference)						1.00 (Reference)
≥ 2.32	0.47	0.20	2.31	0.021	1.59 (1.07 ~ 2.36)		0.43	0.22	1.99	0.047	1.53 (1.01 ~ 2.34)
PLR											
< 196.5					1.00 (Reference)						
≥ 196.5	0.41	0.26	1.60	0.109	1.50 (0.91 ~ 2.48)						
FIB, g/L											
< 3.05					1.00 (Reference)						
≥ 3.05	0.23	0.21	1.13	0.258	1.26 (0.84 ~ 1.89)						
AGR											
< 1.2					1.00 (Reference)						
≥ 1.2	0.18	0.24	0.76	0.447	1.20 (0.75 ~ 1.91)						
ALP, U/L											
< 101					1.00 (Reference)						
≥ 101	0.17	0.21	0.83	0.406	1.19 (0.79 ~ 1.78)						
Treatment											
IC + CCRT					1.00 (Reference)						
IC + CCRT + AC	-0.44	0.32	-1.35	0.176	0.64 (0.34 ~ 1.22)						
IC + CCRT + AC + NTZ/E	-0.43	0.34	-1.28	0.200	0.65 (0.34 ~ 1.26)						
IC + CCRT + NTZ/E	0.25	0.35	0.74	0.462	1.29 (0.65 ~ 2.54)						
IC + RT	-0.49	0.53	-0.92	0.356	0.61 (0.22 ~ 1.73)						
IC + RT + AC	0.18	0.44	0.42	0.676	1.20 (0.51 ~ 2.86)						
IC + RT + AC + NTZ/E	-0.36	0.37	-0.96	0.340	0.70 (0.34 ~ 1.46)						
IC + RT + NTZ/E	-0.16	0.36	-0.44	0.661	0.85 (0.42 ~ 1.73)						

### Risk stratification

**Fig. 3:**
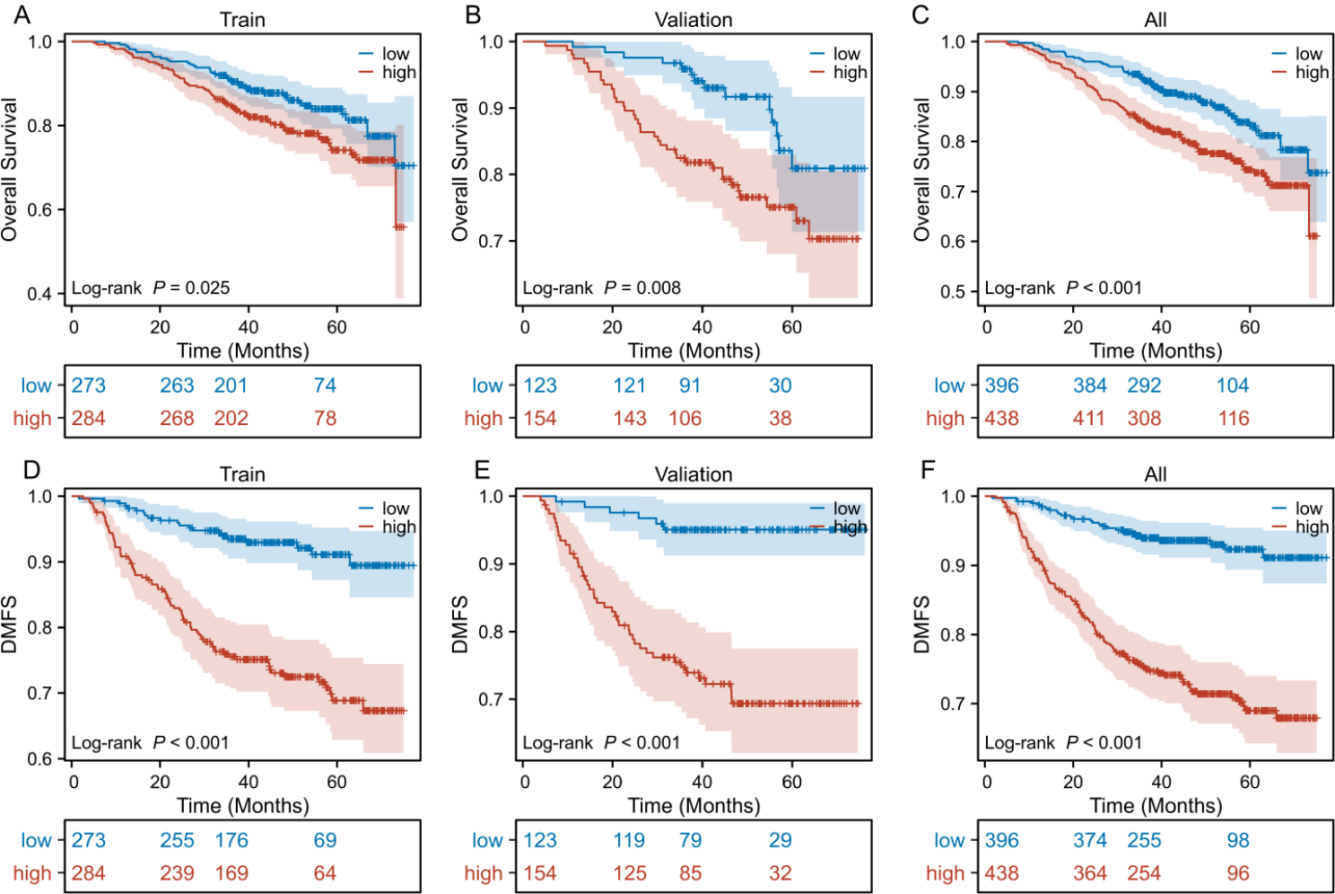
Kaplan-Meier survival curves of different risk groups. OS (**A**) and DMFS (**D**) of the training cohort, OS (**B**) and DMFS (**E**) of the validation cohort, OS (**C**) and DMFS (**F**) of the all. (low-risk group: risk score<149.056; high-risk group: risk score ≥ 149.056)

Utilizing the nomogram, risk scores for each patient with stage IVA NPC were calculated. The optimal cut-off value, determined from the training cohort using a tree model, was identified as 149.056 (Additional file 2). Patients were subsequently stratified into high-risk and low-risk groups based on this threshold. In the training cohort, the 3-year OS rates were 90.8%(95%CI = 0.874–0.943)and 84.8%(95%CI = 0.807–0.891), respectively (*P* = 0.025, Fig. 3A). The 3-year DMFS rates for the low and high-risk subgroups were 93.5%(95%CI = 0.905–0.965)and 75.5%(95%CI = 0.707–0.808), respectively (*P* < 0.001, Fig. 3D).In the validation cohort, the 3-year OS rates were 95.7% and 81.0%, respectively (*P* = 0.008, Fig. 3B).The 3-year DMFS rates for the low and high-risk subgroups were 95.0%(95%CI = 0.912–0.990) and 74.7%(95%CI = 0.681–0.820), respectively (*P* < 0.001, Fig. 3E). Across the entire study population, the 3-year OS rates were 92.4(95%CI = 0.898–0.95)and 84.0%(95%CI = 0.806–0.875), respectively (*P* < 0.001, Fig. 3C).The 3-year DMFS rates for the low and high-risk groups were 94%(95CI%=0.916–0.964)and 75.3%(95CI%=0.713–0.795), respectively (*P* < 0.001, Fig. 3F). These results indicate that the DMFS and OS in the low-risk group were significantly better than those in the high-risk group, further validating the discriminative ability of the model.

### Subgroup analysis of high-risk group

**Fig. 4:**
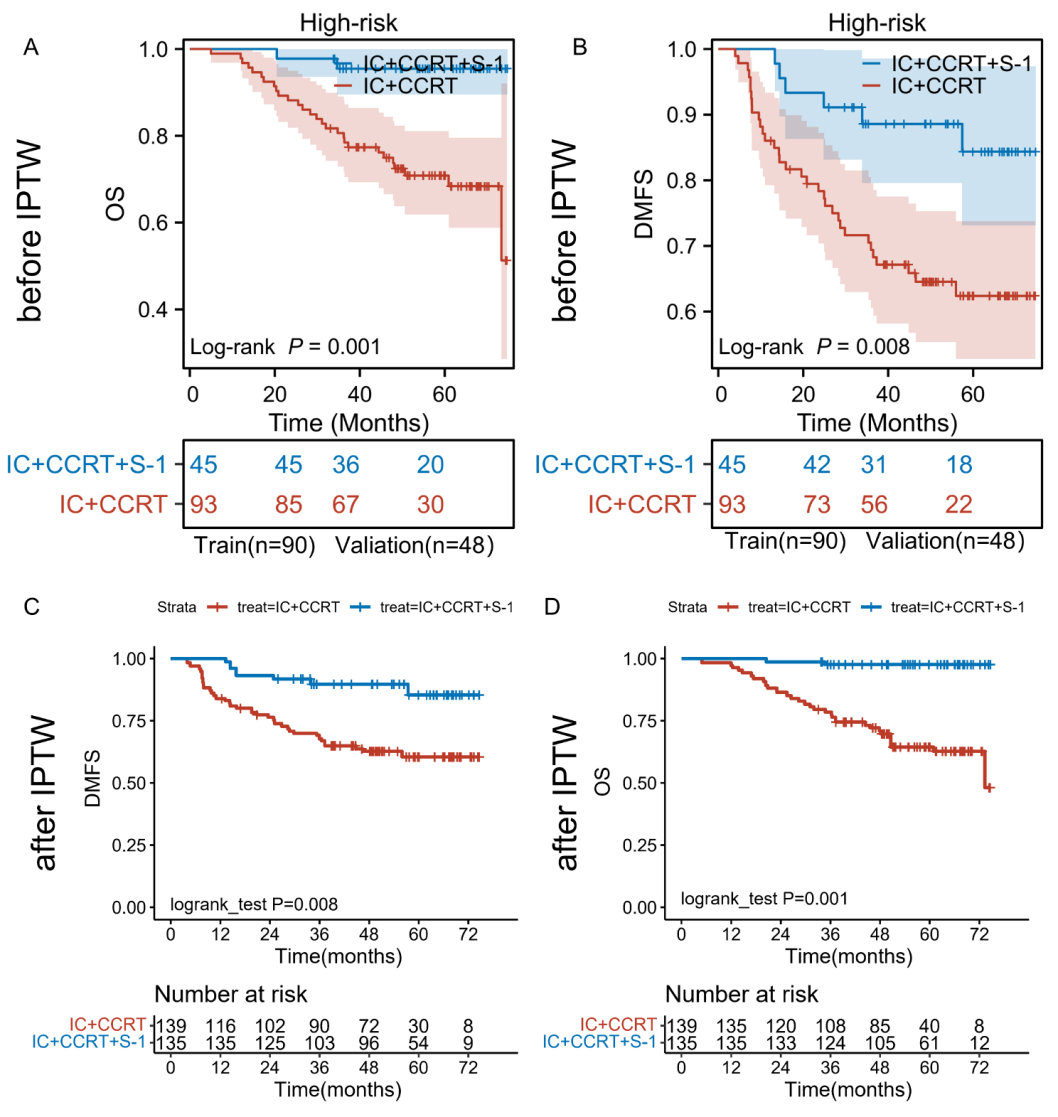
Kaplan-Meier survival curve in high-risk group, (**A**) DMFS before IPTW, (**B**) OS before IPTW, (**C**) DMFS after IPTW, (**D**) OS before IPTW. (IC + CCRT: induction chemotherapy plus concurrent chemoradiotherapy; IC + CCRT + S-1: induction chemotherapy plus concurrent chemoradiotherapy plus S-1 maintenance therapy)

Further analysis of various risk strata revealed distinct outcomes for patients in the high-risk group, as detailed in Table 3; Fig. 4. Within this cohort, patients receiving IC combined with IC + CCRT + S-1 demonstrated significantly improved outcomes compared to those undergoing standard IC + CCRT. Specifically, the 3-year DMFS rates were 88.6% (95% CI = 0.796–0.986) for IC + CCRT + S-1 versus 69.4% (95% CI = 0.605–0.795) for IC + CCRT (*P* = 0.008, Fig. 4A). Similarly, the 3-year OS rates were 95.4% (95% CI = 0.895-1) for IC + CCRT + S-1 compared to 80.6% (95% CI = 0.73–0.891) for IC + CCRT (*P* = 0.001, Fig. 4B). These findings highlight the superiority of the IC + CCRT + S-1 treatment regimen in terms of 3-year DMFS and OS compared to the standard IC + CCRT protocol. Utilizing IPTW to match baseline characteristics (Additional file 3) further validated the clinical significance of S-1 for high-risk individuals. The results revealed that both OS (*p* = 0.001 after IPTW, Fig. 4C) and DMFS (*p* = 0.008 after IPTW, Fig. 4D) benefited from IC + CCRT + S-1 treatment in the high-risk group, irrespective of IPTW adjustment.

**Table 3:** Baseline characteristics of patients in high-risk group

Variables	Total (n = 138)	IC + CCRT (n = 93)	IC + CCRT + S-1 (n = 45)	Statistic	*P*
Gender, n(%)				χ²=1.50	0.221
Female	30 (21.74)	23 (24.73)	7 (15.56)		
Male	108 (78.26)	70 (75.27)	38 (84.44)		
Number of cycles of induction chemotherapy, n(%)				-	0.160
2	18 (13.04)	14 (15.05)	4 (8.89)		
3 ~ 4	115 (83.33)	74 (79.57)	41 (91.11)		
5 ~ 6	5 (3.62)	5 (5.38)	0 (0.00)		
Induction chemotherapy regimens, n(%)				χ²=5.08	0.024
GP	73 (52.90)	43 (46.24)	30 (66.67)		
TP	65 (47.10)	50 (53.76)	15 (33.33)		
Simultaneous chemotherapy regimens, n(%)				-	0.810
Carboplatin	3 (2.17)	2 (2.15)	1 (2.22)		
Nedaplatin	121 (87.68)	82 (88.17)	39 (86.67)		
Lobaplatin	2 (1.45)	2 (2.15)	0 (0.00)		
Cisplatinum	12 (8.70)	7 (7.53)	5 (11.11)		
T stage, n(%)				χ²=4.19	0.381
0	1 (0.72)	0 (0.00)	1 (2.22)		
1	16 (11.59)	10 (10.75)	6 (13.33)		
2	23 (16.67)	17 (18.28)	6 (13.33)		
3	26 (18.84)	15 (16.13)	11 (24.44)		
4	72 (52.17)	51 (54.84)	21 (46.67)		
N stage, n(%)				-	0.055
0	1 (0.72)	1 (1.08)	0 (0.00)		
1	11 (7.97)	10 (10.75)	1 (2.22)		
2	51 (36.96)	38 (40.86)	13 (28.89)		
3	75 (54.35)	44 (47.31)	31 (68.89)		
Number of cycles of synchronised chemotherapy, n(%)				χ²=2.11	0.348
1	24 (17.39)	16 (17.20)	8 (17.78)		
2	93 (67.39)	60 (64.52)	33 (73.33)		
3	21 (15.22)	17 (18.28)	4 (8.89)		
pre EBV DNA (copies/mL), n(%)				χ²=0.28	0.598
< 10,100	33 (23.91)	21 (22.58)	12 (26.67)		
≥ 10,100	105 (76.09)	72 (77.42)	33 (73.33)		
NLR, n(%)				χ²=0.00	0.987
<2.32	52 (37.68)	35 (37.63)	17 (37.78)		
≥ 2.32	86 (62.32)	58 (62.37)	28 (62.22)		
PNI, n(%)				χ²=0.37	0.540
<57.9	127 (92.03)	87 (93.55)	40 (88.89)		
≥ 57.9	11 (7.97)	6 (6.45)	5 (11.11)		
Age (years), n(%)				χ²=0.93	0.336
<60	110 (79.71)	72 (77.42)	38 (84.44)		
≥ 60	28 (20.29)	21 (22.58)	7 (15.56)		

### Subgroup analysis of low-risk group

**Fig. 5:**
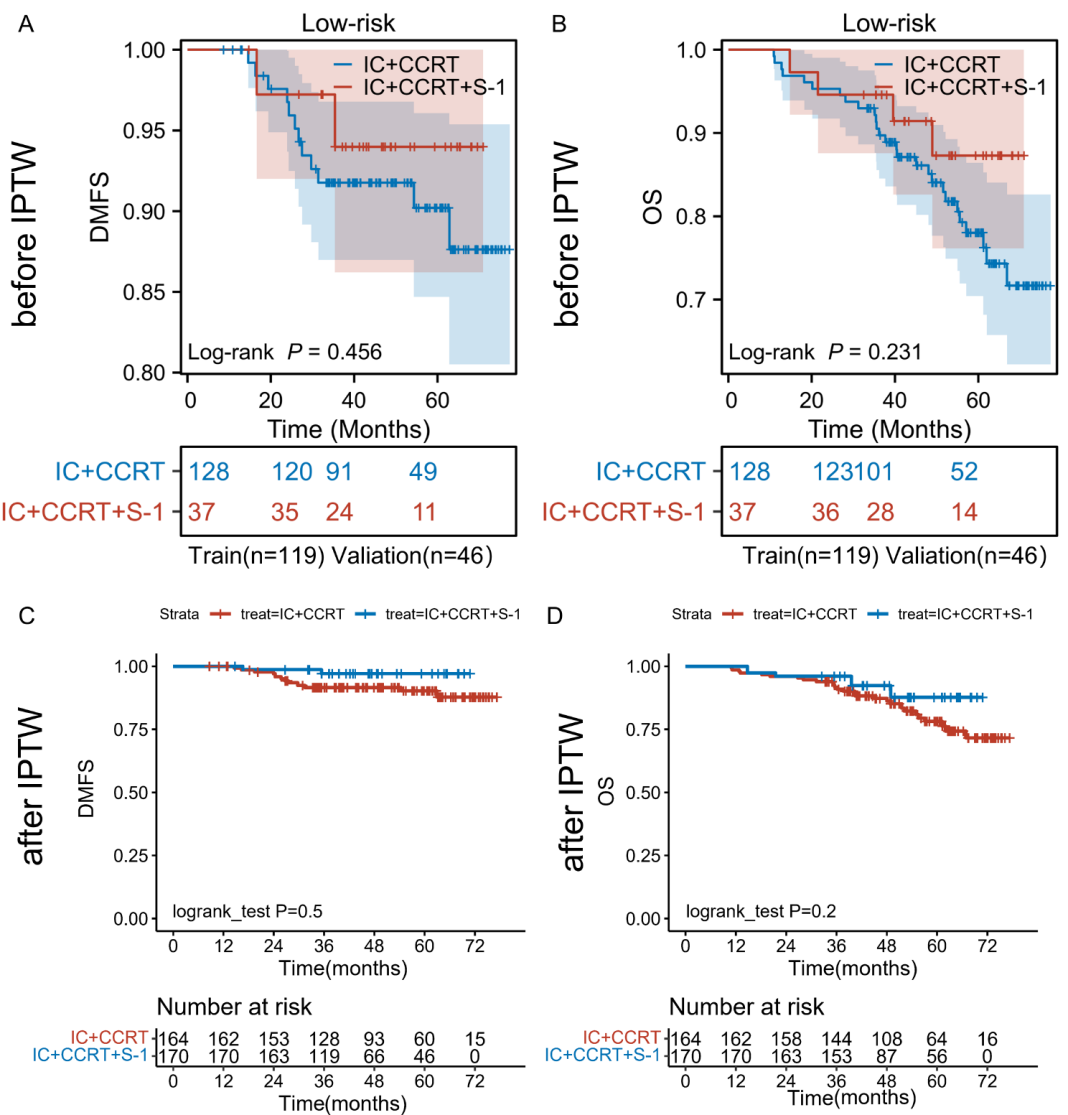
Kaplan-Meier survival curve in low-risk group, (**A**) DMFS before IPTW, (**B**) OS before IPTW, (**C**) DMFS after IPTW, (**D**) OS before IPTW. (IC + CCRT: induction chemotherapy plus concurrent chemoradiotherapy; IC + CCRT + S-1: induction chemotherapy plus concurrent chemoradiotherapy plus S-1 maintenance therapy)

For the low-risk group, baseline characteristics were delineated in Table 4. We conducted a comparative analysis of Disease-Free Survival (DMFS) and Overall Survival (OS) between IC + CCRT and IC + CCRT + S-1, revealing respective 3-year DMFS rates of 91.8% (95% CI = 0.870–0.968) and 94.0% (95% CI = 0.862-1) (*P* = 0.456, Fig. 5A), and 3-year OS rates of 90.5% (95% CI = 0.856–0.958) and 94.6% (95% CI = 0.876-1) (*P* = 0.231, Fig. 5B). Additionally, we employed Inverse Probability of Treatment Weighting (IPTW) to balance baseline characteristics across the population for further confirmation of our findings. Appendix Table 2 illustrates baseline characteristics before and after IPTW adjustment. Notably, regardless of IPTW application, IC + CCRT demonstrated non-inferior 3-year DMFS and OS compared to IC + CCRT + S-1(Fig. 5C-D). Therefore, in the low-risk population, a prudent consideration of treatment intensity reduction, while maintaining therapeutic efficacy, could enhance patient tolerance and mitigate toxicities.

**Table 4:** Baseline characteristics of patients in low-risk group

Variables	Total (n = 165)	IC + CCRT (n = 128)	IC + CCRT + S-1 (n = 37)	Statistic	*P*
Gender, n(%)				χ²=0.74	0.391
Female	40 (24.24)	33 (25.78)	7 (18.92)		
Male	125 (75.76)	95 (74.22)	30 (81.08)		
Number of cycles of induction chemotherapy, n(%)				χ²=1.93	0.380
2	46 (27.88)	39 (30.47)	7 (18.92)		
3 ~ 4	108 (65.45)	81 (63.28)	27 (72.97)		
5 ~ 6	11 (6.67)	8 (6.25)	3 (8.11)		
Induction chemotherapy regimens, n(%)				χ²=8.88	0.003
GP	76 (46.06)	51 (39.84)	25 (67.57)		
TP	89 (53.94)	77 (60.16)	12 (32.43)		
Simultaneous chemotherapy regimens, n(%)				-	0.902
Nedaplatin	142 (86.06)	111 (86.72)	31 (83.78)		
Lobaplatin	4 (2.42)	3 (2.34)	1 (2.70)		
Cisplatinum	19 (11.52)	14 (10.94)	5 (13.51)		
T stage, n(%)				-	0.010
1	6 (3.64)	3 (2.34)	3 (8.11)		
2	15 (9.09)	8 (6.25)	7 (18.92)		
3	12 (7.27)	8 (6.25)	4 (10.81)		
4	132 (80.00)	109 (85.16)	23 (62.16)		
N stage, n(%)				χ²=13.45	0.004
0	15 (9.09)	15 (11.72)	0 (0.00)		
1	91 (55.15)	75 (58.59)	16 (43.24)		
2	26 (15.76)	19 (14.84)	7 (18.92)		
3	33 (20.00)	19 (14.84)	14 (37.84)		
Number of cycles of synchronised chemotherapy, n(%)				χ²=6.52	0.038
1	26 (15.76)	21 (16.41)	5 (13.51)		
2	114 (69.09)	83 (64.84)	31 (83.78)		
3	25 (15.15)	24 (18.75)	1 (2.70)		
Age (years), n(%)				χ²=7.53	0.006
<60	129 (78.18)	94 (73.44)	35 (94.59)		
≥ 60	36 (21.82)	34 (26.56)	2 (5.41)		
pre EBV DNA (copies/mL), n(%)				χ²=0.54	0.464
< 10,100	141 (85.45)	108 (84.38)	33 (89.19)		
≥ 10,100	24 (14.55)	20 (15.62)	4 (10.81)		
PNI, n(%)				χ²=3.96	0.047
<57.9	138 (83.64)	111 (86.72)	27 (72.97)		
≥ 57.9	27 (16.36)	17 (13.28)	10 (27.03)		
NLR, n(%)				χ²=1.10	0.294
<2.32	118 (71.52)	89 (69.53)	29 (78.38)		
≥ 2.32	47 (28.48)	39 (30.47)	8 (21.62)		

## Discussion

In our study, we conducted a retrospective analysis and found that pretreatment plasma EBV DNA, NLR, PNI and N stage are independent risk factors for distant metastasis in stage IVA nasopharyngeal carcinoma (NPC). We developed a nomogram model integrating these four factors to predict the risk of distant metastasis in 834 patients diagnosed with stage IVA NPC.

Notably, T stage did not emerge as a risk factor for distant metastasis, while an increased N stage correlated with a heightened risk of distant metastasis in stage IVA NPC [20]. This study further supports the hypothesis that distant metastasis in NPC typically initiates with lymph node dissemination rather than originating from the primary tumor. Tumor cells exhibit distinct metabolic patterns from normal cells, relying predominantly on glycolysis for energy metabolism even in oxygen-sufficient environments [21].

A low PNI is indicative of a decline in both peripheral blood lymphocyte count and albumin levels, strongly hinting at suboptimal nutritional status and a weakened immune system in patients. A study conducted within the same institution has firmly established the predictive value of PNI in NPC patients [22]. Additionally, a retrospective analysis on newly diagnosed metastatic nasopharyngeal carcinoma patients has further bolstered the argument that PNI serves as a superior nutritional predictor in comparison to BMI [23]. Nevertheless, another investigation revealed that PNI did not emerge as an independent predictive factor when evaluated alongside NRI, CRP, ALP, and LDH [24]. This inconsistency could be attributed to the distinct patient populations targeted by each study.

Elevated levels of plasma EBV DNA strongly correlate with an augmented risk of distant metastasis in regions with a high prevalence of NPC [25] and serve as reliable prognostic indicators. Varied studies demonstrate significant disparities in the optimal cut-off values of pretreatment EBV DNA for survival prediction. Leung et al.’s investigation [26] stratified patients with early-stage NPC into high-risk (similar to stage III survival outcomes) and low-risk (similar to stage I survival outcomes) subgroups, with a designated EBV DNA cut-off value of 4000 copies/mL. Similarly, Lin et al.’s study [27], analyzing 99 patients with locally advanced NPC, revealed that pre-treatment plasma EBV DNA concentrations exceeding 1500 copies/mL were associated with significantly worse OS and locoregional recurrence-free survival (LRFS). In our present study, plasma EBV DNA emerged as an independent predictor of distant metastasis in stage IVA NPC, with a specific cut-off value of 10,100 copies/mL.

The inflammatory response of the organism profoundly influences tumorigenesis, development, metastasis, and prognosis. The Neutrophil-to-Lymphocyte Ratio (NLR), as an inflammation indicator, mirrors the tumor immune microenvironment, closely correlating with the prognosis of various malignant tumors [28–32]. NLR stands as a crucial marker for prognostic prediction in NPC patients and aids TNM staging for effective prognostic assessment. A high NLR level suggests heightened tumor aggressiveness and a predilection for distant metastasis, resulting in an unfavorable prognosis. Stage IVA NPC, characterized by unsatisfactory outcomes despite standard treatment modalities, has been classified in previous studies into three types via the TNM staging system: type A (predominantly nasopharyngeal primary foci), type D (predominantly regional lymph node metastasis), and type AD (both). In this study, the multidimensional assessment of the risk of distant metastasis in stage IVA NPC involved combining clinically important factors to achieve precise treatment.

Simultaneous radiotherapy followed by oral maintenance chemotherapy emerges as an elective therapeutic option for patients with locally advanced NPC. In this study, we observed that IC + CCRT + S-1 significantly enhanced DMFS and OS compared to IC + CCRT in the high-risk scoring group. Conversely, in the low-risk scoring group, IC + CCRT + S-1 did not yield superior DMFS and OS outcomes. This implies the necessity of maintenance therapy for stage IVA NPC in the high-risk scoring group, while low-risk patients may benefit from close clinical observation to avert over-treatment, thereby offering a valuable reference for the precise treatment of stage IVA NPC. Our study aligns with the objectives and conclusions of previous research, which aimed to identify high-risk groups that would benefit from adjuvant chemotherapy, with the goal of achieving personalized and less toxic treatment. However, our study distinguishes itself by incorporating a broader range of easily accessible clinical variables (e.g., PNI, NLR, EBV DNA) to enhance predictive accuracy, focusing specifically on stage IVA patients, and employing distinct endpoints, namely the occurrence of distant metastasis [18].

Inevitably, several limitations persist in retrospective studies. Firstly, inherent selectivity bias is unavoidable due to the retrospective nature of the study. Additionally, the availability of information on patient-specific characteristics is constrained by limitations in data sources. Secondly, the study was confined to data from a single center, necessitating validation with a diverse cohort from different centers to confirm the accuracy of the findings. Thirdly, potential bias may arise from variations in the choice of treatment regimen among different clinicians. Fourthly, we cannot guarantee the complete similarity in the distribution of relevant features between the training and validation sets. Finally, the applicability of the nomogram model to younger patients (< 18 years old) and regions with lower NPC incidence demands further validation. In terms of the specific limitations mentioned in the study, for patients with stage IVA/B NPC, the study indicates that the TPC induction regimen is more effective than the PF induction regimen, supporting the TPC regimen as the new standard regimen for induction chemotherapy in NPC [33]. The induction regimens for patients in our study were primarily GP and TP. Due to potential biases arising from individual heterogeneity, we did not include more personalized induction chemotherapy regimens. In future studies, we look forward to further promoting the TPC regimen’s clinical application and gradually improving and refining our predictive model.

## Conclusions

Pre EBV DNA, NLR, PNI and N stage emerged as independent risk factors for DMFS in stage IVA NPC. In the context of IMRT, the nomogram developed in this study exhibits commendable accuracy and discriminative prowess in predicting distant metastasis for stage IVA NPC. Importantly, our nomogram can guide the utility of S-1 adjuvant chemotherapy, offering crucial guidance for selecting optimal treatment modalities for patients at high and low risk of distant metastasis in stage IVA NPC.

### Electronic supplementary material

Below is the link to the electronic supplementary material.

Supplementary Material 1

## Data Availability

No datasets were generated or analysed during the current study.

## Abbreviations

NPC: Nasopharyngeal carcinoma
IMRT: Intensity modulated radiation therapy
EBV: Epstein-Barr virus
LDH: Lactate dehydrogenase
NLR: Neutrophil to Lymphocyte Ratio
PLR: Platelet to Lymphocyte Ratio
Fib: Fibrinogen
AGR: Albumin to Globulin Ratio
NTZ: Nimotuzumab
E: Endostar
IC: Induction Chemotherapy
CCRT: Concurrent chemoradiotherapy
CC: Concurrent chemotherapy
AC: Adjuvant chemotherapy
DMFS: Distant metastasis-free survival
OS: Overall Status
ROC: Receiver operating curve
PNI: Prognostic Nutritional Index
