# Supplementary material for: A nomogram based on nutritional and inflammatory parameters to predict DMFS and identify beneficiaries of adjuvant chemotherapy in IVA-stage nasopharyngeal carcinoma
Source: BMC Cancer. 2024 May 11;24:578. doi: 10.1186/s12885-024-12330-6 (PMC11088054; doi:10.1186/s12885-024-12330-6)
Supplement: Supplementary file 1 — Supplementary Material 1 [file 12885_2024_12330_MOESM1_ESM.docx]

## Multimedia Appendix

**Additional file 1**

Determination of EBV DNA, LDH, NLR, PLR, AGR, and Fib

Before treatment, we used real-time quantitative polymerase chain reaction (RT-qPCR) to detect patients' plasma EBV DNA levels. The assay kit was purchased from Sun Yat-sen University Daan Genetics Co Ltd (Guangzhou, China). Each plasma specimen was subjected to DNA extraction by a magnetic bead cassette (EA20160201; PerkinElmer) and an automated nucleic acid extraction workstation (Pre-NAT; PerkinElmer), and then the RT-qPCR system was used to amplify DNA fragments in the BamHI-W fragment region of the EBV genome and to measure the concentration of circulating EBV DNA.ABI Prism7500 sequence detector was used to acquire data and analyses were performed using the Sequence Detection System (version 1.6.3; Applied Biosystems), with multiple blank water controls included in each analysis. Results were expressed as the number of copies of the EBV genome per millilitre of plasma, with 0 copies/ml recorded if plasma EBV DNA was not detected by qPCR.

Pre-treatment serum LDH levels were detected by an enzyme kinetic kit (manufactured by Roche, Germany) using a ModularPP fully automated biochemical analyser, and the normal reference range for serum LDH levels was 120 to 250 IU/L.

The ratio of neutrophil count to lymphocyte count for pre-treatment haematology routine was defined as NLR, the ratio of platelet count to lymphocyte count was PLR, and AGR was the ratio of albumin to globulin. Pre-treatment haematological routine test indices, such as neutrophil count, lymphocyte count, platelet count, and pre-treatment routine biochemical test indices, such as albumin, globulin, and albumin-globulin ratio indices, were measured by XN-9000 haematology analyser and ModularPP fully automated biochemical analyser; pre-treatment Fib as fibrinogen was measured by coagulation analyser, and the normal value was 2.0 to 4.0 g/L. The rest of the haematological indices were stratified according to the standard of Fujian Provincial Cancer Hospital.

**Additional file 2** Tree model of risk score. (The optimal cut-off value of the risk score based on the nomogram model was 149.056)


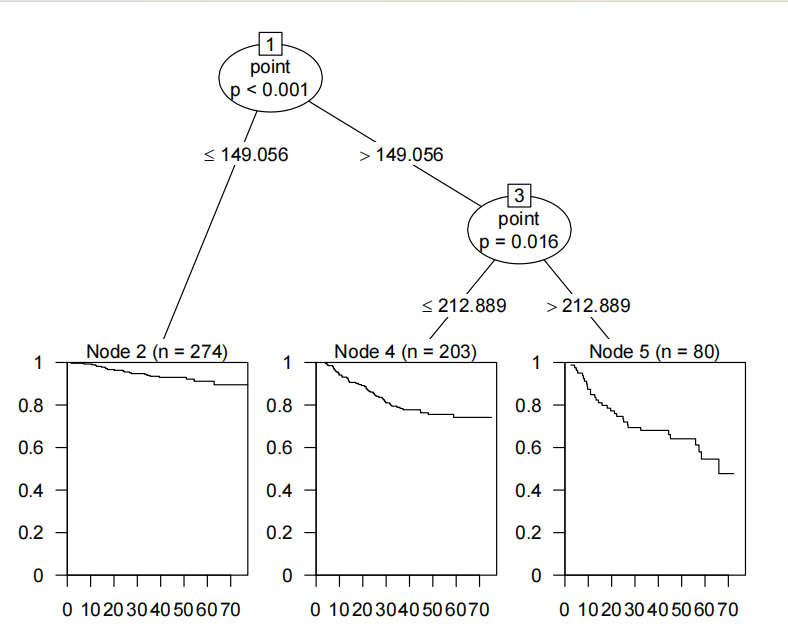


**
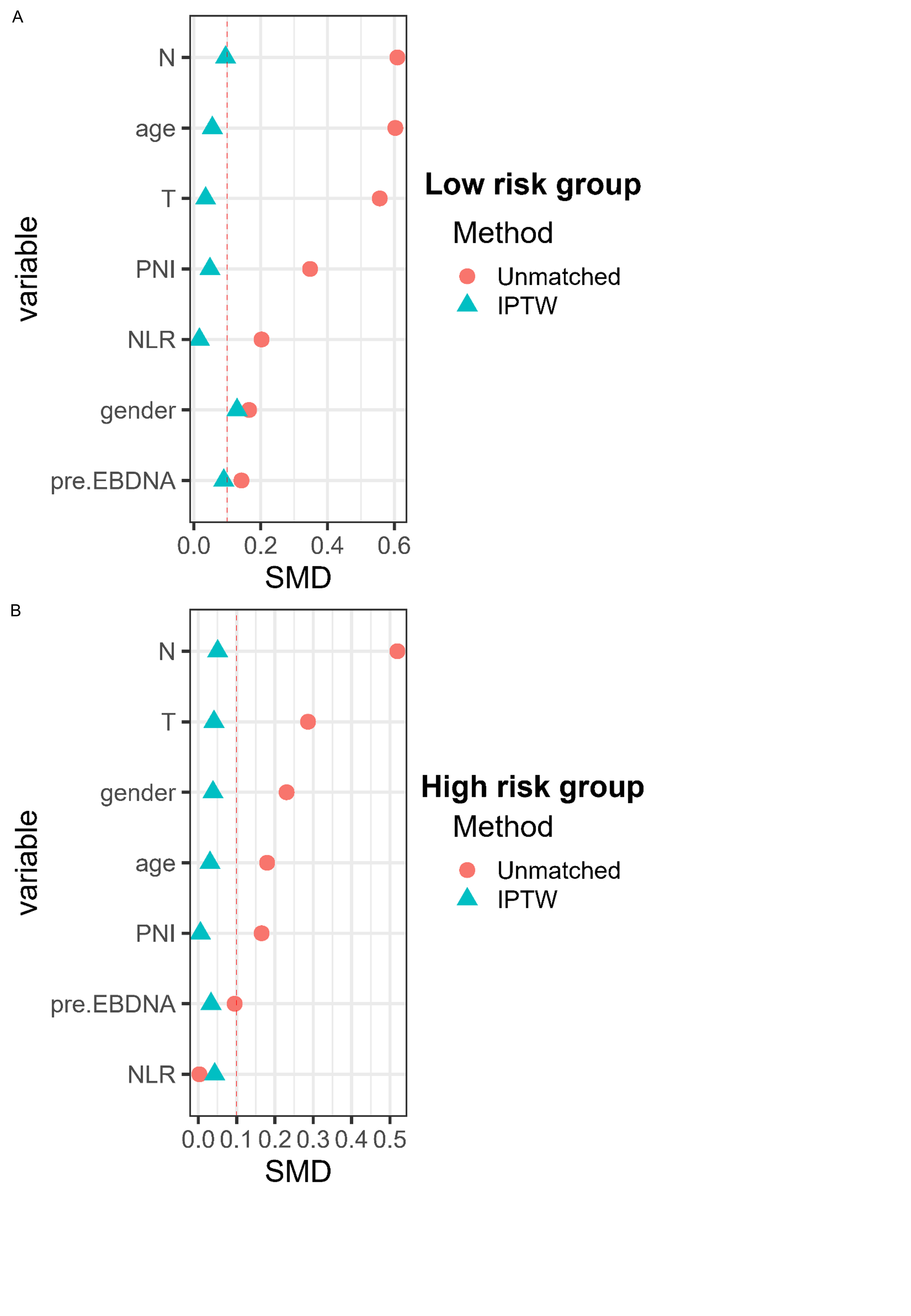
Additional file 3** Figure A: Love plot before and after inverse probability of treatment weighting (IPTW) among the low-risk group.Figure B: Love plot before and after inverse probability of treatment weighting (IPTW) among the low-risk group.

**Additional file 4** Baseline table before and after inverse probability of treatment weighting (IPTW) among the low-risk group.

| Variables |  | Unmatched NA NA | | P | IPTW NA NA | | P |
| --- | --- | --- | --- | --- | --- | --- | --- |
|  |  | IC+CCRT+S-1 n=128 | IC+CCRT n=37 |  | IC+CCRT+S-1 n=164.3 | IC+CCRT n=169.7 |  |
|  |  |  |  |  |  |  |  |
| gender (%) | Female | 33 (25.8) | 7 (18.9) | 0.522 | 40.3 (24.5) | 32.6 (19.2) | 0.542 |
|  | Male | 95 (74.2) | 30 (81.1) |  | 124.0 (75.5) | 137.1 (80.8) |  |
| age (%) | ＜60 | 94 (73.4) | 35 (94.6) | 0.012 | 128.3 (78.1) | 128.5 (75.7) | 0.864 |
|  | ≥60 | 34 (26.6) | 2 ( 5.4) |  | 36.0 (21.9) | 41.2 (24.3) |  |
| T (%) | 0-1 | 3 ( 2.3) | 3 ( 8.1) | 0.015 | 5.4 ( 3.3) | 5.4 ( 3.2) | 0.997 |
|  | 2 | 8 ( 6.2) | 7 (18.9) |  | 14.6 ( 8.9) | 14.0 ( 8.3) |  |
|  | 3 | 8 ( 6.2) | 4 (10.8) |  | 12.8 ( 7.8) | 14.6 ( 8.6) |  |
|  | 4 | 109 (85.2) | 23 (62.2) |  | 131.6 (80.1) | 135.7 (80.0) |  |
| N (%) | 0-1 | 90 (70.3) | 16 (43.2) | 0.004 | 106.2 (64.7) | 115.0 (67.8) | 0.871 |
|  | 2 | 19 (14.8) | 7 (18.9) |  | 25.3 (15.4) | 20.7 (12.2) |  |
|  | 3 | 19 (14.8) | 14 (37.8) |  | 32.7 (19.9) | 34.0 (20.0) |  |
| pre EBVDNA (%) | ＜10100 | 108 (84.4) | 33 (89.2) | 0.641 | 140.2 (85.4) | 150.0 (88.4) | 0.679 |
|  | ≥10100 | 20 (15.6) | 4 (10.8) |  | 24.1 (14.6) | 19.7 (11.6) |  |
| PNI (%) | ＜57.9 | 111 (86.7) | 27 (73.0) | 0.082 | 137.9 (83.9) | 145.4 (85.6) | 0.789 |
|  | ≥57.9 | 17 (13.3) | 10 (27.0) |  | 26.4 (16.1) | 24.4 (14.4) |  |
| NLR (%) | ＜2.32 | 89 (69.5) | 29 (78.4) | 0.399 | 117.8 (71.7) | 120.4 (71.0) | 0.947 |
|  | ≥2.32 | 39 (30.5) | 8 (21.6) |  | 46.5 (28.3) | 49.3 (29.0) |  |

**Additional file 5** Baseline table before and after inverse probability of treatment weighting (IPTW) among the high-risk group.

| Variables |  | Unmatched NA NA | | P | IPTW NA NA | | P |
| --- | --- | --- | --- | --- | --- | --- | --- |
|  |  | IC+CCRT+S-1 n=128 | IC+CCRT n=37 |  | IC+CCRT+S-1 n=164.3 | IC+CCRT n=169.7 |  |
|  |  |  |  |  |  |  |  |
| gender (%) | Female | 33 (25.8) | 7 (18.9) | 0.522 | 40.3 (24.5) | 32.6 (19.2) | 0.542 |
|  | Male | 95 (74.2) | 30 (81.1) |  | 124.0 (75.5) | 137.1 (80.8) |  |
| age (%) | ＜60 | 94 (73.4) | 35 (94.6) | 0.012 | 128.3 (78.1) | 128.5 (75.7) | 0.864 |
|  | ≥60 | 34 (26.6) | 2 ( 5.4) |  | 36.0 (21.9) | 41.2 (24.3) |  |
| T (%) | 0-1 | 3 ( 2.3) | 3 ( 8.1) | 0.015 | 5.4 ( 3.3) | 5.4 ( 3.2) | 0.997 |
|  | 2 | 8 ( 6.2) | 7 (18.9) |  | 14.6 ( 8.9) | 14.0 ( 8.3) |  |
|  | 3 | 8 ( 6.2) | 4 (10.8) |  | 12.8 ( 7.8) | 14.6 ( 8.6) |  |
|  | 4 | 109 (85.2) | 23 (62.2) |  | 131.6 (80.1) | 135.7 (80.0) |  |
| N (%) | 0-1 | 90 (70.3) | 16 (43.2) | 0.004 | 106.2 (64.7) | 115.0 (67.8) | 0.871 |
|  | 2 | 19 (14.8) | 7 (18.9) |  | 25.3 (15.4) | 20.7 (12.2) |  |
|  | 3 | 19 (14.8) | 14 (37.8) |  | 32.7 (19.9) | 34.0 (20.0) |  |
| pre EBVDNA (%) | ＜10100 | 108 (84.4) | 33 (89.2) | 0.641 | 140.2 (85.4) | 150.0 (88.4) | 0.679 |
|  | ≥10100 | 20 (15.6) | 4 (10.8) |  | 24.1 (14.6) | 19.7 (11.6) |  |
| PNI (%) | ＜57.9 | 111 (86.7) | 27 (73.0) | 0.082 | 137.9 (83.9) | 145.4 (85.6) | 0.789 |
|  | ≥57.9 | 17 (13.3) | 10 (27.0) |  | 26.4 (16.1) | 24.4 (14.4) |  |
| NLR (%) | ＜2.32 | 89 (69.5) | 29 (78.4) | 0.399 | 117.8 (71.7) | 120.4 (71.0) | 0.947 |
|  | ≥2.32 | 39 (30.5) | 8 (21.6) |  | 46.5 (28.3) | 49.3 (29.0) |  |
